# Derivation of Empirical Relationships to Predict Cambodian Masonry Strength

**DOI:** 10.3390/ma15145030

**Published:** 2022-07-20

**Authors:** Nurmurat Kandymov, Nor Fazilah Mohd Hashim, Syuhaida Ismail, Serdar Durdyev

**Affiliations:** 1 Razak Faculty of Technology and Informatics, Universiti Teknologi Malaysia, Kuala Lumpur 54100, Malaysia; syuhaida.kl@utm.my; 2 Department of Architectural and Engineering Studies, Ara Institute of Canterbury, Christchurch 8011, New Zealand; durdyevs@ara.ac.nz

**Keywords:** brick, mortar, compressive strength, masonry, prism, Cambodia

## Abstract

Masonry material characteristics, such as compressive strength, and the relationship between brick, mortar, and masonry compressive strengths are required for the analysis and assessment of masonry structures. This paper aimed to investigate the compressive strength relationship of Cambodian masonry. A total number of 42 prisms were constructed in the laboratory using six different brick/mortar combinations. Two brick types, solid and hollow, and three—1:3, 1:4, and 1:6—cement–sand mortar combinations were used. The effects of brick and mortar strengths were analysed. Using regression analysis, a simple empirical relationship was derived for masonry compressive strength as a function of brick and mortar strength in the Cambodian context.

## 1. Introduction

Brick masonry is a common building construction option in the world due to the low cost of materials, good insulation, and a simple construction process. Masonry is constructed using individual bricks and mortar; thus, assemblage properties are affected by both brick and mortar properties [1]. Compressive strength of masonry is an important parameter for the structural design of masonry. The relative strength of units and mortar, shape of the units, mortar joint thickness, and testing type of specimen are factors that affect the behaviour of masonry under an axial compression test [1,2,3,4]. Gumaste et al. [5] relate masonry strength to brick strength, directly stating that masonry strength is 25–50% of brick strength, while other researchers, namely Murthi et al. and Padalu and Singh [6,7] found that masonry compressive strength increases with increasing brick strength. Thaickavil and Thomas [8] found other additional parameters pertinent to masonry compressive strength, slenderness ratio of prism, volume fraction of masonry unit, and volume ratio of bed joint to mortar. In general, the strength of the brick masonry will increase proportionally with brick/mortar strengths [5].

There are different ways to obtain mechanical properties for existing masonry buildings: in situ field tests, laboratory tests on specimens extracted from the field, and testing prisms constructed in the laboratory [9]. With flat-jack in situ tests conducted by researchers [10], however, the results are generally not precise enough for structural design. It is not always possible to extract masonry prism samples to conduct compression tests due to the structure type, function, and more importantly cost. Moreover, removing and transporting them to the laboratory without any damage constitute another challenge [7]. Therefore, researchers [1,5,8,11,12,13,14] attempted to develop an empirical relationship between brick, mortar, and masonry compressive strength to predict the compressive strength of masonry by mortar and brick unit strengths. However, there is a significant difference, geographically, in both material properties and proportion of the cement in mortars adopted in the construction industry. For instance, the compressive strength of bricks used in Western countries’ brick masonry is between 15 and 150 MPa, while in India the value is between 3 and 20 MPa [6,7,8,11,12,14,15,16,17,18,19,20,21,22,23]. These differences in properties of brick and mortar indicate the urgent need for the derivation of the empirical relationship for brick masonry regionally.

Therefore, this paper attempts to investigate properties of brick masonry prisms under axial compression using the solid and hollow local bricks of Cambodia with different ratios of mortars. Results of compressive strength will then be used to derive coefficients in order to predict Cambodian brick masonry strength by brick and mortar strengths.

## 2. Past Studies on Coefficient Values for Masonry Strength

Compression testing of masonry prisms is not always practical; therefore, attempts have been made in the literature for developing different empirical models to predict masonry compressive strengths. One of the earliest prediction models was proposed by Engesser [24], which suggested the algebraic sum of brick and mortar strengths with certain proportions. Another unique prediction model was suggested by Roca et al. [25] in which masonry strength depended more on mortar strength. Dymiotis and Gutlederer [26] derived a second-order polynomial equation and suggested a formula after regression analysis, which accounts for mortar and brick compressive strength. Dayaratnam [27] suggested a model in which the same weightage was given to bricks and mortar with the same constant parameters, while Roza [28] and Thaickavil and Thomas [8] proposed more sophisticated models with more variables in which the relative volume of the unit, the relative volume of mortar, and the slenderness ratio of the prism are considered, in addition to brick and mortar strengths.

Most of the researchers [1,5,11,13,14,19,29,30,31,32,33,34,35,36] suggested a model based on brick and mortar compressive strengths using the set of constants K, *α*, and *β*. The relationship is of the form shown in Equation (1) [37]:

f’_m_ = K f_b_^*α*^ × f_j_^*β*^ (1)

where K, *α*, and *β* are constants, and f_b_, f_j_, and f’_m_ are masonry unit (brick), mortar, and masonry compressive strength, respectively. Eurocode 6 [37] recommends a variety of K values depending on the brick-unit properties and bond configuration, where *α* and *β* are described as 0.7 and 0.3, respectively.

Lumantarna et al. [1] developed the coefficient to predict masonry compressive strength from brick and mortar for New Zealand unreinforced masonry. They investigated both existing masonry structures and laboratory-based constructed prisms. There were 45 prisms extracted and 75 were built in the laboratory from 14 brick–mortar combinations. Mortar grades consisted of cement, lime, and sand with different water ratios. The highest compressive strength mortar was one with the highest cement proportions, where five specimens were tested for each mortar grade. The field-extracted sample test results were combined with laboratory-based samples, and nonlinear regression analysis was carried out with *DataFit 9.0* software (Oakdale Engineering 2010, Oakdale, PA, USA). The software examiness the resemblance between the field-extracted and laboratory-constructed samples and, according to the results of their study, they converged. The coefficients they found for K, *α*, and *β* were 0.75, 0.75, and 0.31, respectively.

Kaushik et al. [14] investigated the stress–strain characteristics of clay bricks in the Indian context. Prisms from combinations of four brick and three mortar types were investigated, and each prism type tested seven specimens for the accuracy of the results. Coefficient values for K, *α*, and *β* were found to be 0.63, 0.49, and 0.32, respectively. Coefficient of determination (R^2^) and standard error for estimate (σ) were also calculated for the derived empirical relationships. The coefficient of determination shows the reliability of the relationship between experimentally obtained values and regression analysis. On the other hand, the standard error for estimates provide an idea of the scatter of actual data from the value estimated by linear regression analysis. For empirical coefficients, the values R^2^ and σ were found to be 0.93 and 0.48 MPa, respectively.

In addition, Gumaste et al. [5] studied the empirical relationship with both prisms and wallettes in the Indian context. Two types of bricks—table moulded and wire cut—and five different mortar grades were used. Unlike other studies, [1,14,19], soil was also used in some mortar grades. Combinations were used for stack and English bonded prisms and wallettes. Coefficients were found to be 0.317, 0.866, and 0.134 for the stack bond, and 0.225, 0.855, and 0.146 for English bond-type walls for K, *α*, and *β*, respectively.

Christy et al. [13] studied short prisms of 3.63 h/t ratio under axial compressive test for clay brick and fly ash brick. A weak mortar of a 1:6 ratio was selected and fine aggregate was replaced with fly ash of 0, 10, and 20% in order to examine the effect of fly ash on prism bond. Prisms were also reinforced with woven wire mesh at alternate bed joints, and the results were compared among reinforced and unreinforced prisms. In all specimens, reinforced prisms obtained greater compressive strength values compared to unreinforced prisms, and those differences decreased while fly ash value in mortar increased from 0% to 20%. Coefficients were derived to predict masonry compressive strength and E of the prisms, and K, *α*, and *β* values were found to be 0.35, 0.65, and 0.25, respectively.

Thamboo and Dhanasekar [19] investigated correlations between the performance of solid prisms and wallettes in the Sri Lankan masonry context. Five different clay and compressive earth bricks and two mortar proportions were used to test 50 prisms and 40 wallettes under compression testing. Prism compressive strength was higher than wallette compressive strength, but a linear relationship was found between prisms and wallettes. Nonlinear regression analysis was conducted to predict the masonry compressive strength for prism and wallettes separately. K, *α*, and *β* were found to be 0.2, 1.26, and 0.15, and 0.25, 1.09, and 0.12 for prisms and wallettes, respectively.

Coefficients derived in the literature to predict masonry strength are provided in Table 1. The common property of those studies is that the value of constant *α* is greater than constant *β*, which means that masonry strength depends more on brick strength than mortar strength. Only few researchers [12,30] found greater value for *β*, indicating that the prism strength relies more on mortar strength. Only Dayaratnam [27] suggested equal weights for both brick and mortar, while Basha and Kaushik [12] found very little effects of brick and mortar strengths by suggesting a K value over 1.

## 3. Experimental Programme/Laboratory Testing

### 3.1. Materials/Brick–Mortar Properties

**Figure 1:**
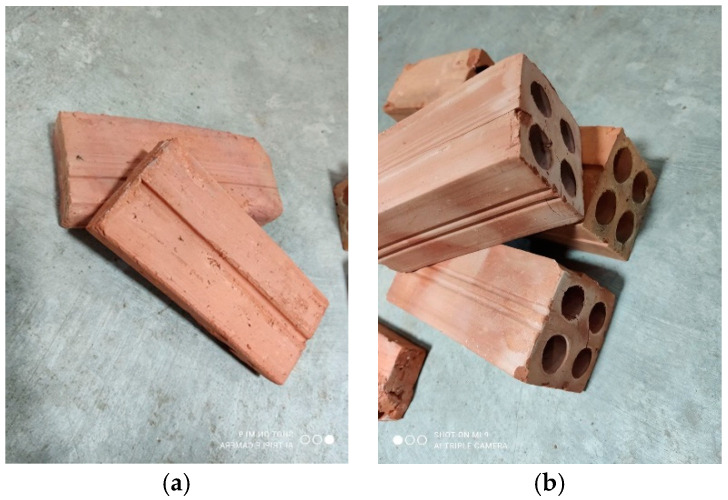
(**a**) Solid clay brick; (**b**) hollow clay brick.

Two types of masonry units and three types of mortar are used to build the masonry prisms. The bricks used in this research are solid and hollow hand-moulded clay bricks, as shown in Figure 1. The length and width are the same for both types, 170 and 75 mm, respectively, where the height is 75 mm for hollow and 30 mm for solid bricks. These bricks are purposely selected to cover the compressive strengths of commonly used brick types in the construction industry in Cambodia. The compressive strength of the units is determined as per BS EN 772-1:2011 [38], and 10 specimens were tested to determine the strength of each unit type. Some standards [39] do not allow an investigation of compressive strength in a sample that has an h/t ratio of less than 0.4, and solid and hollow brick samples fit the requirement with ratios of 0.4 and 1, respectively. All specimens are loaded on the 170 mm × 75 mm area.

**Figure 2:**
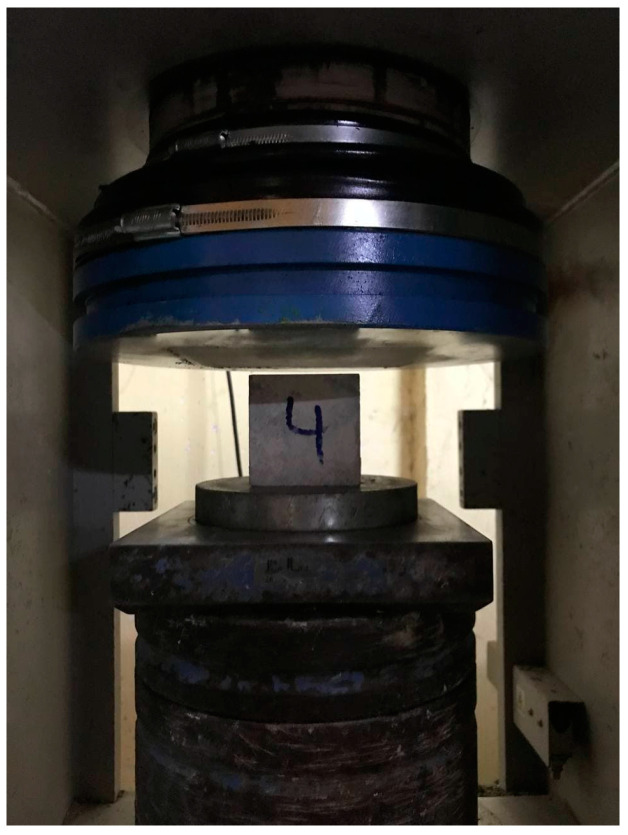
Mortar compression test.

In this study, three types of mortar with mix proportions are used to construct the prisms in combination with two types of brick units. Portland cement-to-sand ratios of 1:3, 1:4, and 1:6 were used for mortar mixing. Strong mix mortar, 1:3, is denoted as A; weak, 1:6, is denoted as B, whereas the most-adopted ratio in the construction industry of Cambodia, 1:4, is denoted as type C in the following discussion. Dry sand and cement are mixed first, and then water is added to the dry mixture in the ratios of 0.7 and 1.1 for types A and B, respectively. For type C, the ratio at which water is added is based on the experience of the mason who is assisting in the preparation process of cubes. Six cube samples of 70 mm size were taken from each mortar mixture and were left for 28 days curing at room temperature in order to prepare for compression testing. The compressive strength of the mortars is determined as per the ASTM C109 [40] standard (Figure 2).

### 3.2. Testing Procedures

**Figure 3:**
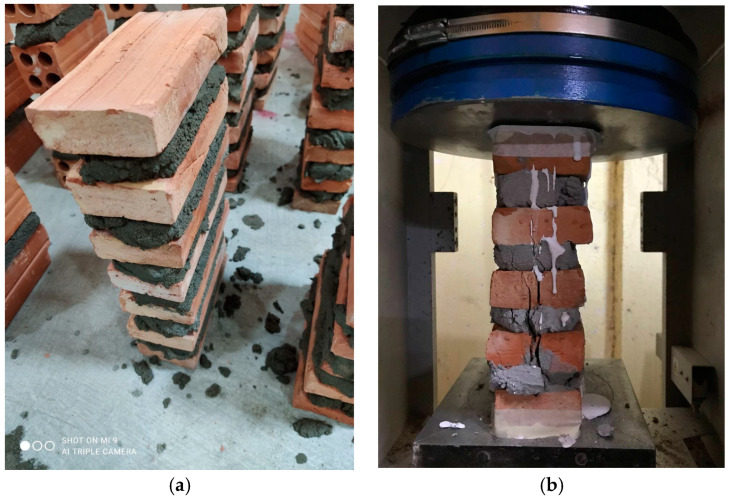
(**a**) Solid brick prism; (**b**) Prism failure.

In this study, a total of 42 prisms with 6 different brick/mortar combinations were prepared with occasional assistance provided by an experienced mason, and they were cured for 28 days at room temperature before assessing the masonry strength. Length and height conform to the ASTM standard, which are a minimum of 100 mm length and a minimum of 2 units of height, with height in the lowest dimension between 1.3 and 5, respectively [41]. Complete test matrix and specimen dimensions are provided in Table 2 below. Seven prisms were built for each of the six combinations. Four to seven bricks are used in each prism depending on the brick type (Figure 3), as the heights of solid and hollow bricks are different.

Mortar thickness in both prisms is kept at a constant of 20 mm to replicate the common Cambodia unreinforced masonry construction practice. All bricks are kept in water for 10 min before construction, as suggested by Sarangapani et al. [18], to avoid poor brick–mortar bond because of dry bricks.

**Figure 4:**
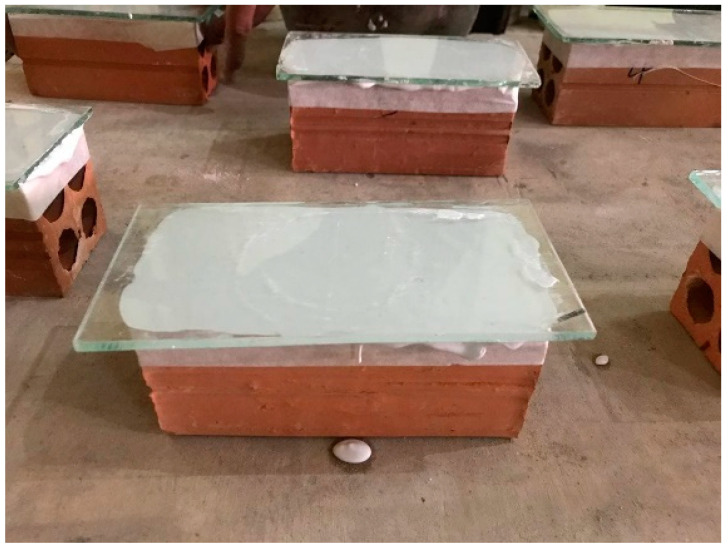
Capping of bricks.

All bricks and prisms are capped using a gypsum plaster in order to make a smooth surface so that the applied load can be distributed equally (Figure 4). In addition, specimens were aligned carefully between platens to avoid eccentricity in loading. Prisms were tested in accordance with ASTM C1314-16 [42] using the 2000 kN universal testing machine.

## 4. Results and Discussion

The compressive strength of the bricks was determined as explained in the previous section under uniaxial compression loading. In hollow bricks, f_b_ varied from 8.7 to 12.1 MPa, with a mean value of 10.2 MPa (COV 10.3%), while in solid bricks, the value varied from 61.2 to 94.1, with a mean value of 77.8 MPa (COV 15%), as shown in Table 3. Differences in compressive strength between the two brick types were enormous, and this could be due to the physical properties and production technology. Height and h/t ratio are primary physical properties affecting the strength of units with an inverse relationship [43]. The height of the hollow brick was 2.5 times higher than the solid brick and h/t values were 1 and 0.4 for hollow and solid bricks, respectively. Moreover, holes in hollow bricks are another reason for the compressive strength difference between brick types, as the holes cause lesser compressive strengths [43]. Another physical property for the compressive strength difference is density. Solid brick density is almost twice the density of hollow brick, and there is a direct relationship between the density and compressive strength of the units [44]. The type of production and burning temperature are two factors affecting the compressive strength in production technology. Extruded and moulded production types were selected according to the plasticity of brick, and there is a strong relationship between compressive strength and production type [43]. The burning temperature of bricks varies between 700 and 1100 °C, and it has strong relationship with the compressive strength of the brick units [43,45]. The production type and burning temperature of the bricks during the production process were not within the scope of the paper due to budget and time constraints. Therefore, causes of these two properties in compressive strength difference are not known. Loading area of the specimen and roughness of the surface are other factors affecting compressive strength [43]. However, the loading area is the same for both units, and they were capped with gypsum plaster.

Table 4 shows the mortar unit properties for all three types of mortars: weak (1:6), intermediate (1:4), and strong (1:3). For weak mortar, compressive strengths varied from 3.8 to 5.9 MPa, with an average strength of 4.81 (COV 14%). Intermediate mortar-type compressive strength varied from 9.1 to 12.1 MPa, with an average compressive strength of 10.56 MPa (COV 12.4%). Strong mortar obtaineded the highest value, as was expected, ranging from 12.3 to 15.3 MPa, with an average compressive strength of 13.8 MPa (COV 8.3%). The highest compressive strength value of intermediate-type mortar was less than the lowest value of strong mortar. In addition, the coefficient of variation was the lowest in strong mortar specimens. The density of the mortar types also demonstrated a linear relationship with the compressive strength of the respective types.

Masonry prisms of six combinations from two brick and three mortar types were tested for compressive strength. Table 5 shows the results for all combinations, which were found to be in good agreement with studies from the literature [5,7,8,12,14,15,21,22]. The mean value of f’_m_ was found to be 1.99 MPa (COV 11.9%), 2.01 MPa (COV 9%), and 2 MPa (COV 9%) for hollow brick prisms constructed with *weak*, *intermediate*, and *strong* mortar, respectively. The coefficient of variation values was also around 10%, which is quite acceptable. The mean value of f’_m_ was found to be 4.31 MPa (COV 13%), 8.11 MPa (COV 10.7%), and 8.23 MPa (COV 12.9%) for solid brick prisms constructed with *weak*, *intermediate*, and *strong* mortar, respectively. The coefficient of variation values for all three groups was between 10.7% and 13%, which is good and acceptable. The lowest value for solid brick compressive strength was obtained from weak mortar prisms, as expected, and the value was twice that of the hollow brick prism strength. Padalu and Singh [7] illustrated, using a graph, that the compressive strength of a prism increased with mortar strength. However, hollow brick prisms demonstrated the same results for compressive strength for all three mortar types. The reason for this observation may be due to the bond strength between brick and mortar. The debonding of the brick–mortar interface decreases the effectiveness of load transfers through the mortar layer [7], and the surface of the hollow brick is very smooth, which significantly affects the brick–mortar bond. Moreover, prisms tend to bulge laterally when the axial load is applied with the top and bottom parts of the specimen under compression with confinement pressure, while the middle zone is subjected to tension [7]. Masonry is weak in resisting tension forces [46] and has poor brick–mortar bonding with respect to hollow brick prisms due to the smooth surface; hollow brick prisms resulted in poor performance with all three mortar ratios. Another reason for compressive strength differences between brick and hollow prisms is due to the bricks’ unit strength, as Padalu and Singh [7] found a clear relationship between brick unit strength and prism strength. Additionally, the later expansion of mortar under compressive loading is restricted due to the frictional forces at the brick–mortar interface, and mortar may resist direct load transfer and attributes and increase in prism strength [7]. Prism compressive strengths, factored in accordance with ASTM C1314 [42] based on the prism height to least lateral dimension, are also provided in Table 5 below. The correction factors are calculated by linear interpolation since the h/t ratio of the prisms lies between the given values in the ASTM standard [42].

### Estimation of Prism Strength of Masonry

**Figure 5:**
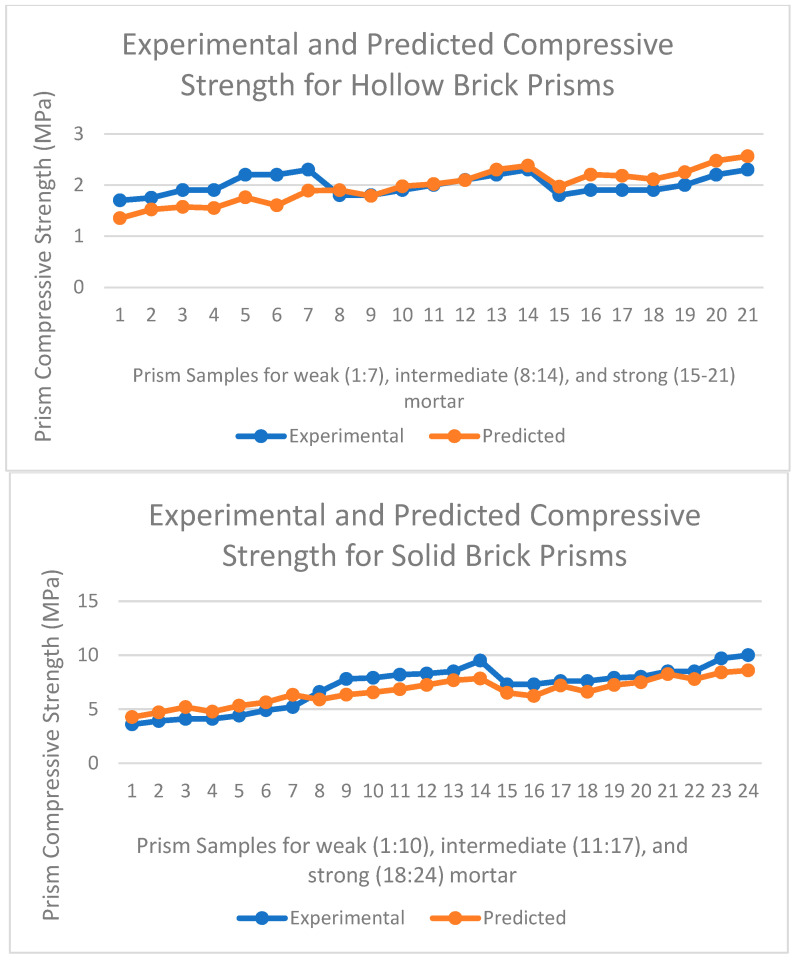
Experimental and predicted compressive strength values for hollow and solid brick prisms.

Compressive strength is the fundamental property of the masonry and is useful in a variety of ways, including the design of masonry walls, the estimation of E_m_, and plotting of stress–strain curves. Thus, f’_m_ is the most essential property and must be always known for masonry. Nevertheless, it is not always possible to conduct compression testing of prisms due to feasibility or cost factors. On the other hand, compressive strengths of brick and mortar are available in codes or can be easily conducted. Therefore, these three compressive strengths can be efficiently related, as is performed in Eurocode [37] and in other studies [1,5,11,12,13,14,19,29,30,31,32,33,34,35,36]. According to the regression analysis conducted with software R and in agreement with previous studies [1,5,11,12,13,14,33,35,36], the α value (0.59) was found to be greater than the β value (0.32), which implied that masonry compressive strength does not depend as much on mortar strength as it does on brick strength. In other words, mean mortar compressive strength had less influence on mean masonry compressive strength compared to mean brick compressive strength. In addition, the K value was found to be 0.24. The derived coefficient values provided results similar to those of recent studies in the literature [11,13,14,29]. The latest six studies’ [1,11,12,19,30,32] proposed coefficients were applied to the present data, and masonry compressive strength was predicted with those coefficients from the respective studies. The coefficient of determination was calculated for each prediction, and the R^2^ results obtained were a very poor match, with values of −71, −243, −79, −158, −12, and 0.29. The results support the objective of deriving the coefficients of empirical relationships for brick masonry regionally. Experimentally obtained and predicted compressive strength values are depicted separately for each type of prism in Figure 5.

R^2^ is the coefficient of determination between experimentally obtained values and the value obtained by regression analysis, and the results, which are close to unity, indicate a good fit for the obtained analysis. σ is the standard error of estimate that provides an idea of the scatter of actual data from the value estimated by regression analysis. It is desirable that σ is at the minimum value, meaning that scatter in the data with respect to the estimated value is at the minimum [14]. In the present study, Equation (1) had a coefficient of determination (R^2^) and standard error of estimate (σ) values of 0.94 and 0.78 MPa, respectively, which were deemed satisfactory and acceptable.

## 5. Conclusions

The compressive strength of masonry is an important parameter for the structural design of masonry, and there are different methods for obtaining this property of masonry, namely, in situ field tests, laboratory tests on specimens extracted from the field, and testing prisms that are constructed in the laboratory [9]. However, there are some disadvantages and limitations in the first two methods [7,10]. Therefore, researchers [1,5,11,12,13,14,19,29,30,31,32,33,34,35,36] attempted to develop an empirical relationship to predict masonry strength from brick and mortar strengths, However, there is considerable difference geographically in material properties [5,6,7,8,11,12,14,15,16,17,18,19,20,21,22,23], and these differences indicate that there is a need of derivation of these coefficients for each region. The objective of the study was to determine coefficients to predict masonry compressive strength with masonry constituents of brick and mortar in the Cambodian context. This paper presents the experimental investigation of masonry prisms under uniaxial compression testing. In the laboratory, 42 prisms were constructed using the six brick–mortar combinations. Two masonry unit types, solid and hollow, and three mortar types were used for the prism construction. Experimental results were used to develop empirical relationships to predict the compressive behaviour of brick masonry using only brick and mortar compressive strengths in order to assist designers in detailed analysis. The following summary can be drawn from this research:

- Significant variation has been observed in compressive strength of hollow and solid bricks. Physical properties, such as hole, height, and density, and production technology such as burning temperature could constitute the difference in variation of the compressive strength.
- Mechanical properties of masonry and its constituent materials were obtained by uniaxial compression testing according to the respective standards. The average compressive strengths of the masonry prisms constructed with 1:3, 1:4, and 1:6 cement-sand mortar were found to be in good agreement with past studies in the literature [5,7,8,12,14,15,21,22].
- The mean masonry compressive strength was found to increase with the increase in brick compressive strength, which was found to be in good agreement with previous researchers [7].
- It has been observed that increase in mortar strength increases the masonry’s compressive strength in solid bricks, in agreement with the literature [7]. However, that effect could not been observed in hollow bricks, and this observation could be due to the weak brick–mortar bond of hollow bricks, which has very smooth surfaces.
- A predictive equation relating brick and mortar compressive strengths with masonry compressive strength in the form of the Eurocode 6 [37] expression was derived, and constants were found to be 0.24, 0.59, and 0.32 for K, *α*, and *β*, respectively.
- Brick constant (*α*) in the prediction equation was found to be greater than the mortar constant (*β*), in agreement with past studies [1,5,11,12,13,14,19,33,35,36], and it indicates that the brick units have more influence on masonry compressive strength compared to mortar strength.

Lastly, the bricks used in this study were only from two manufacturers, and both of them are located in the capital city. More comprehensive research with different manufacturers from a variety of locations in the country is recommended, since the types of brick production method, burning temperature, clay used as raw material, etc., affect the mechanical properties of the brick. Moreover, a limited number of samples were tested for the compression test. Therefore, derived coefficients cannot be generalized to all Cambodian masonry due to the limited number of samples and limited brick manufacturer variety.

## Figures and Tables

**Table 1:** Coefficients for specific models in the literature to predict masonry compressive strength.

Research	Year	K	*α*	*β*
[19]	2019	0.2	1.26	0.15
[32]	2019	0.25	1.03	0.28
[30]	2018	0.1	0.34	1.93
[11]	2016	0.69	0.6	0.35
[12]	2014	1.34	0.1	0.33
[1]	2014	0.75	0.75	0.31
[13]	2013	0.35	0.65	0.25
[29]	2012	0.61	0.51	0.36
[14]	2007	0.63	0.49	0.32
[5]	2007	0.32	0.86	0.13
[5]	2007	0.22	0.85	0.14
[27]	1987	0.27	0.5	0.5
[33]	1986	0.32	0.53	0.21
[35]	1982	0.83	0.66	0.33
[34]	1973	0.9	0.67	0.33
[36]	1963	0.68	0.5	0.33

**Table 2:** Test matrix and dimensions of the specimens.

Type	Name	Brick Type	Mortar Type	h (mm)	l (mm)	w (mm)
Prism	S3	Solid	A	360	170	75
Prism	H3	Hollow	A	370	170	75
Prism	S4	Solid	C	360	170	75
Prism	H4	Hollow	C	370	170	75
Prism	S6	Solid	B	360	170	75
Prism	H6	Hollow	B	370	170	75

**Table 3:** Brick unit properties (f_b_).

Unit Type	Dimension (L × W × H) (mm)	Density/(kg/m^3^) (ASTM C90-16a)	Compressive Strength/(MPa) (BS EN 772-1:2011)
Hollow (10 specimens)	170 × 75 × 75	1256 (10.4%)	10.20 (10.3%)
Solid (10 specimens)	170 × 75 × 30	2484 (7.7%)	77.75 (15%)

**Table 4:** Mortar unit properties (f_j_).

Mortar Type (Cement: Sand)	Density/(kg/m^3^) BS EN 1015-10	Compressive Strength/(MPa) ASTM C109
A (1:3)	1895 (0.5%)	13.8 (8.3%)
B (1:6)	1783 (1.6%)	4.81 (14%)
C (1:4)	1870 (1.7%)	10.56 (12.4%)

**Table 5:** Summary of Test Results for Masonry Prisms (f’_m_).

Brick Type	f’_m_ (MPa)	Normalised f’_m_ (MPa)
Prisms with weak mortar (1:6)		
Hollow	1.99 (11.9%)	2.41
Solid	4.31 (13%)	5.19
Prisms with intermediate mortar (1:4)		
Hollow	2.01 (9%)	2.44
Solid	8.11 (10.7%)	9.78
Prisms with strong mortar (1:3)		
Hollow	2 (9%)	2.42
Solid	8.23 (12.9%)	9.92

## Data Availability

The data presented in this study are available on request from the corresponding author. The data are not publicly available due to privacy.
